# Platelet count and coagulation profiles of adult hypertensive patients at Felege Hiwot comprehensive specialized hospital, Northwest Ethiopia: A comparative cross-sectional study

**DOI:** 10.1371/journal.pone.0329022

**Published:** 2025-08-12

**Authors:** Mitku Kassa, Yemataw Gelaw, Mesay Arkew, Haftu Asmerom, Kabtamu Gemechu, Elias Shiferaw

**Affiliations:** 1 Department of Medical Laboratory Sciences, Felege Hiwot Comprehensive Specialized Hospital, Bahir Dar, Ethiopia; 2 Department of Hematology and Immunohematology, School of Biomedical and Laboratory Sciences, College of Medicine and Health Sciences, University of Gondar, Gondar, Ethiopia; 3 School of Medical Laboratory Sciences, College of Health and Medical Sciences, Haramaya University, Harar, Ethiopia; Sai Gosavi Specialty Clinic / Nano Hospitals Bangalore / Saraswati Specialty Clinic, INDIA

## Abstract

**Background:**

Hypertension is a global public health problem and associated with metabolic, cellular, and blood disturbances. Hematological and hemostatic disturbance have been documented in individuals with hypertension, playing a pivotal role in hypertension associated vascular complications. Despite this, there is a scarcity of evidence and reports regarding the coagulation profiles are contradicting among hypertensive patients. Thus, this study aimed to compare the platelet count and coagulation profiles of adult hypertensive patients with those of normotensive controls at Felege Hiwot Comprehensive Specialized Hospital in Northwest Ethiopia, from June to August, 2023.

**Methods:**

A comparative cross-sectional study was conducted among a total of 180 study participants (120 hypertensive patients and 60 healthy individuals) recruited by consecutive sampling technique. Socio-demographic, clinical and anthropometric data were collected using structured questionnaires and checklist. A total of six milliliters of venous blood (3 ml in EDTA tube and 3 mL in citrate tube) was collected for platelet count and coagulation profile determination. Platelet count and coagulation profile were determined using Uni Cel DxH800 hematology analyzer and Huma cue due plus coagulation analyzer, respectively. One-way ANOVA, Kruskal Wallis test, and correlation analysis was used during data analysis. A p-value of <0.05 was considered statistically significant.

**Results:**

In the present study, the median (interquartile range) age was 42.5 (36.0–50.0) years for newly diagnosed, 50.0 (45.0–65.0) years for treatment and 40.0 (35.0–45.7) years for the control group (P < 0.001). The overall hemostatic abnormality in hypertensive patient was 83 (69.2%), and significant difference was found in the median of activated partial thromboplastin time, prothrombin time and international normalized ratio between hypertensive patients as compared to control. Statistically significant difference was observed in the median (interquartile range) of activated partial thromboplastin time (P = 0.041), prothrombin time (P < 0.001), and international normalized ratio (P < 0.001) results between newly diagnosed hypertensive patients and healthy controls. Moreover, there was also a significant difference in activated partial thromboplastin time (P = 0.020), prothrombin time (P = 0.011), and international normalized ratio (P = 0.012) results between hypertensive patients undergoing treatment and healthy controls.

**Conclusions:**

Hemostatic abnormality was observed in majority of hypertensive patients. Compered to healthy controls, the median values of activated partial thromboplastin time, prothrombin time and international normalized ratio were significantly higher in hypertensive patients. This finding highlights the importance of assessing coagulation parameters in hypertensive patients in order to prevent complications related to abnormal blood clotting. However, further longitudinal studies are necessary to gain better understanding of the changes in coagulation observed in hypertensive patients.

## Introduction

Hypertension (HTN) is the state of having high blood pressure (blood pressure of ≥140 mmHg systolic or ≥90 mmHg diastolic or on medication) [1]. Worldwide, the number of people living with HTN doubled between 1990 and 2019, from 650 million to 1.3 billion [2]. Uncontrolled HTN is the leading cause of cardiovascular disease and premature death across the globe [3]. Although it is a preventable and controllable risk factor for cardiovascular disease, many developing nations have not yet given it that much attention [4]. The rate of HTN increases with age and it is highly prevalent among adults aged 30–79 years, with two third of them residing in low- and middle-income nations [1, 2]. Recent epidemiological data indicate that the burden of hypertension accounts for 36% in Africa [1], and 25.9% in sub-Saharan Africa [5]. In Ethiopia, it is estimated that about 19.6% of the population has hypertension [6]. A study conducted in Bahir Dar city indicated that 24.3% of the population has hypertension and of these, 44.4% are unaware of having the condition [7].

Hypertension is a silent disease and it is diagnosed at a delayed stage in the majority of patients when complications develop and require immediate treatment [8, 9]. Despite the fact that lowering blood pressure is essential in the treatment of hypertension, it is also crucial to effectively control other cardiovascular risk factors such as dyslipidemia, diabetes, glucose intolerance, obesity, smoking as well as hematological and coagulation disturbances [10, 11]. Both cellular and non-cellular components can contribute to the viscosity, volume, and coagulability of the blood which significantly influence blood pressure regulation [12]. Platelet parameters and basic coagulation profile tests including prothrombin time (PT), activated partial thromboplastin time (APTT) and international normalized ratio (INR) are used to evaluate systemic inflammatory response, hemostatic function, identify coagulopathies, and monitor anticoagulant therapy in different group of patients [13–19].

Hemostatic abnormality indicated by change in PT and APPT was observed in patients with diabetes [20, 21], heart disease [22], bleeding diathesis [14] and HIV/AIDS [23]. Moreover, it has been documented that a significant change was observed in platelet count and coagulation profiles such as PT, APTT, and INR in hypertensive patients as compared to normotensive individuals [24–28]. In addition, the PT and APTT of hypertensive patients’ show a positive correlation between systolic blood pressure and duration of hypertension [27]. Thus, assessment of these parameters at the earlier stage is crucial for preventing severe complication of hypertension due to hemostatic dysfunction.

Hemostatic dysfunction primarily thrombotic coagulopathy play a key role in the development of atherosclerosis, which is the most common complications of hypertension, resulting in an increased morbidity and mortality [29, 30]. It has been reported that hypertensive patients have an increased risk of deep vein thrombosis, pulmonary embolism, and stroke, which are all related to abnormal blood clotting [31]. The pathophysiological change of hemostasis and/or coagulation irregularities in patients with hypertension is thrombogenic rather than hemorrhagic [32–36]. In the light of several evidences, patients with hypertension have shown endothelial dysfunction, platelet hyperactivation, dysregulation of the coagulation and fibrinolytic pathways, which help to promote the induction and the maintenance of prothrombotic state [37–42]. Additionally, evidences from empirical studies suggests that larger platelets are escape from the bone marrow due to increased consumption at the site of a damaged blood vessel, which accelerates thrombotic diseases such as coronary artery disease, cerebrovascular illness [39], and myocardial infarction [38, 43].

Generally, hematologic and hemostasis disturbances are major changes in hypertension, with significant contribution to morbidity and mortality. In Ethiopia, the current guidelines on the management of chronic medical conditions including hypertension don’t include routine assessment of hematological and coagulation parameters unless the patients experience serious complications [22, 44]. Although some studies on the coagulation profiles of hypertension patients have been done in different corner of the world, they came up with a range of contradictory findings. Some studies showed that patients with hypertension have significantly higher value of PT [24-28, 40], APTT [24, 25] and, INR [24], while other studies reported no significant difference in APTT between the hypertensive and normotensive groups [26, 40]. Regarding the result of platelet count, few studies reported that there was significant increase in platelet count among hypertensive patients compared to normotensive group [25, 28], while others reported there is no statically significant difference in platelet count between the groups [45, 46]. To the best of our knowledge, no published studies exist in Ethiopia in particular and the coagulation profile of hypertensive patients is not well studied in general. To assure this claim, common indexation databases such as PubMed/Medline, Web of Sciences, and Scopus was searched. Therefore, this study was intended to compare platelet count and coagulation profile among treatment naïve and treatment experienced hypertensive patients with apparently healthy normotensive group at Felege Hiwot Comprehensive Specialized Hospital, Northwest Ethiopia.

## Materials and methods

### Study design, period, and area

A comparative cross-sectional study was carried out from June to August 2023 at Felege Hiwot Comprehensive Specialized Hospital (FHCSH). The hospital is found in Bahir Dar city, located 565 km northwest to Addis Ababa, the capital city of Ethiopia at an average elevation of 1840 meters [47]. The hospital provides comprehensive healthcare services to a catchment population of more than 7 million. The hospital has been providing teaching, research, and community service. At the time of the study, the hypertension follow-up clinic at Hospital gives service to more than 665 hypertensive patients on regular follow‒up.

### Study participants

This study encompasses three distinct groups: All adult hypertensive patients attending at the chronic care clinic of FHCSH, consisting drug naïve group, who are not currently receiving any anti-hypertensive treatment; the drug experienced group, comprising hypertensive patients who are actively undergoing anti-hypertensive treatment; and the control group, which includes individuals serving as nonremunerated voluntary blood donors at the Bahir Dar blood bank were involved in the current study. On the other hand, hypertensive patients with documented bleeding and clotting disorders, patients who were taking anticoagulant therapy, patients with a known chronic condition like heart, liver, or kidney disease, patients with secondary hypertension, pregnant women, patients diagnosed with diabetes, hepatitis B or C, tuberculosis, HIV/AIDS, hematological malignancies, and patients below the age of 18 and above 65 years were excluded from participation. The health condition of the control groups was evaluated according to the national blood bank service blood donor questionnaire, and screening was done for transfusion-transmitted infectious diseases (HIV, Hepatitis, Syphilis, and malaria).

### Operational definition

Coagulation abnormalities: is an abnormality of one of the hemostasis compartments; thrombocytopenia, abnormal high PT/INR or APTT.

Thrombocytopenia**:** is defined as a platelet count lower than 150 x10^3^ cells/µl [48].

Prolonged PT: is described by a coagulation time that surpasses 16 seconds [49].

Prolonged APTT: is identified as a coagulation time that exceeds 36 seconds [49].

**Abnormal high INR**: INR value of greater than 1.2 [49]

### Sample size and sampling technique

The determination of the sample size was based on the commonly accepted principle of the rules of thumb, which advises a minimum of 30 participants per group to effectively detect differences between groups with a statistical power of 80% [50]. To bolster the statistical robustness and reliability, we decided to increase the number of participants by twofold. This decision resulted in a total of 180 participants being included in the study. Specifically, this total comprised 60 drug-naïve hypertensive patients, 60 drug-experienced hypertensive patients, and 60 normotensive controls. The study participant was recruited consecutively until the desired total number of participants was achieved.

### Data collection and laboratory methods

Information pertaining to socio-demographic and behavioral characteristics was collected using pre-tested structured questionnaire. These data were collected through face-to-face interviews conducted by a trained nurse, ensuring consistency and reliability in the data collection process. Clinical, blood pressure and anthropometric data were collected using structured checklist. Blood pressure (BP) was taken by clinical nurses using an analog sphygmomanometer. Measurements were taken from the upper arm with the hand at the heart level after the patient had been rest for more than 5 minutes. The data regarding anthropometric variables such as height (to the nearest centimeter without shoes), and weight (to the nearest 0.1 kg), were collected according to the anthropometric measurements protocol. Body mass index (BMI) was computed by dividing the weight in kilograms by the square of the height in meters, providing a standardized measure of participants’ body composition [51].

Six-milliliter (ml) of venous blood specimen (3 ml in K2EDTA tube and 3 ml in sodium citrate tube) was collected from the median cubital vein of each study participant by medical laboratory professionals by adhering to standardized operational procedures. Following the collection process, platelet count was analyzed using Unicel DXH 800 (Beckman Coulter, USA) automated hematology analyzer by employing impedance principle [52]. The remaining 3 ml of blood in 3.2% sodium citrate tube was used for assessment of the coagulation profiles from platelet poor plasma (PPP). Coagulation profiles including PT, INR and APTT were determined using the Huma cue due plus coagulation analyzer, by following the turbidity meter principle [53].

### Data quality assurance and management

The English version of questionnaire was translated to local language, Amharic, and then translated back to English to check the consistency. A pre-test was conducted on 5% of the sample size at Tibebe Gione Comprehensive Specialized University Hospital to ensure the validity of the questionnaires. Training was given for the data collectors prior to data collection. Blood pressure and anthropometric measurements was measured twice during each assessment, and the average of the two readings was recorded for analysis. Manufacturer instructions and standard operating procedures were strictly followed during specimen collection and all other laboratory procedures. Blood in K2EDTA and sodium citrate tube was gently mixed 6–8 times to prevent blood clotting. Visual inspection of the collected blood samples was conducted, with gentle mixing to observe for clots as an indicator of sample quality. Daily internal quality control procedures were performed for the hematology and huma cue due plus analyzer. Wright-stained peripheral blood film examination was performed to figure out any suspected pseudo thrombocytopenia. Completion, and clarity of the collected data were checked regularly and the results were properly recorded, transcribed, and reviewed.

### Data analysis and interpretation

The data underwent thorough cleansing and completeness checks, entered into Epi Info version 7 and subsequently exported to SPSS version 25 software for analysis. The normality of data distribution was assessed using the Shapiro wilk test and Kolmogorov-Smirnov test. The results were reported as frequency and percentages for categorical variables, mean ± SD for normally distributed continuous variable and median with interquartile range (IQR) for continuous variables with skewed distribution. Chi-square test were used to determine statistical differences for categorical variables between the study groups. One-way ANOVA and Kruskal-Wallis test supplemented by pairwise post hoc analysis were used to compare platelet count and coagulation profiles between the groups. On the other hand, correlation tests were utilized to examine the correlation of platelet and coagulation profile with BMI and duration of treatment. P < 0.05 determining statistical significance for all analyses.

### Ethical consideration

The study was carried after ethical clearance was secured from the Research and Ethical Review Committee of the School of Biomedical and Laboratory Science, College of Medicine and Health Sciences, University of Gondar, with reference number SBMLS/508. An official permission letter was granted from Felege Hiwot Comprehensive Specialized Hospital, and informed written consent was obtained from each participant prior to data collection. Participants were thoroughly briefed on the study’s potential risks and benefits, and their right to withdraw at any stage. Confidentiality of participant information was strictly maintained, and cases involving coagulation abnormalities as well as individuals screening positive for malaria, syphilis, HIV and hepatitis were promptly communicated to physicians for appropriate management.

## Results

### Socio-demographic, clinical, and anthropometric characteristics of study participant

A total of 180 study participants, including 120 hypertensive patients which comprises 60 newly diagnosed, and 60 patients who are on treatment, as well as 60 healthy individuals as a control group, were enrolled in this study. The median (IQR) age was 42.5 (36.0–50.0) years for newly diagnosed, 50.0 (45.0–65.0) years for treatment and 40.0 (35.0–45.7) years for the control group (P < 0.001). In terms of sex distribution, there were no significance difference across the study groups. Significant difference was observed in the median (IQR) of systolic and diastolic BP among the study groups (P < 0.001). Regarding the anthropometric measurement, there were no significance difference in BMI across the study groups (P = 0.358). (**Table 1**).

**Table 1:** Socio-demographic, clinical characteristics and anthropometric measurements of the study participants at Felege Hiwot Comprehensive Specialized Hospital, Bahir Dar, Northwest Ethiopia, 2023, (n = 180).

Variables		NDx (n = 60)	TRx (n = 60)	Controls (n = 60)	P-value
Age (years), median (IQR)		42.0 (36.0-50.0)	49.0 (42.0-56.0)	40.0 (35.0-45.7)	<0.001*
Sex, n (%)	Male	29 (16.1)	28 (15.6)	30 (16.7)	0.935
	Female	31 (17.2)	32 (17.8)	30 (16.7)	
Residence, n (%)	Urban	47 (26.1)	48 (26.7)	56 (31.1)	0.05
	Rural	13 (7.2)	12 (6.7)	4 (2.2)	
Educational status, n (%)	Unable to read & write	22 (12.2)	27 (15)	2 (1.1)	<0.001*
	Attend primary school	9 (5)	6 (3.3)	4 (2.2)	
	Attend secondary school	9 (5)	9 (5)	15 (8.3)	
	Attend higher education	20 (11.1)	18 (10)	39 (21.7)	
Marital status, n (%)	Single	10 (5.6)	2 (1.1)	9 (5)	0.107
	Married	50 (27.8)	59 (32.8)	50 (27.8)	
Occupation, n (%)	Student	3 (1.7)	–	–	<0.001*
	Government employed	17 (9.4)	10 (5.6)	33 (18.3)	
	Private employed	6 (3.3)	18 (10)	–	
	Farmer	14 (7.8)	13 (7.2)	2 (1.1)	
	Merchant	7 (3.9)	7 (3.9)	13 (7.2)	
	House wife	13 (7.2)	13 (7.2)	11 (6.1)	
Systolic BP, median (IQR)		152.5 (146.0-165.0)	129.5 (122.0-144.7)	115.5 (112.3-118.0)	<0.001*
Diastolic BP, median (IQR)		89.5 (85.0-94.7)	79.0 (74.3-85.0)	76.0 (74.0-78.0)	<0.001*
Duratio]n of HTN (years), mean ± SD		–	4 (3-10)	–	
BMI (kg/m^2^), mean ± SD		24.7 ± 3.75	24.8 ± 3.85	24.02 ± 1.7	0.358

BMI, Body Mass Index; BP, Blood pressure; HTN, Hypertension; IQR, Interquartile range; SD, Standard Deviation, NDx, Newly Diagnosed; TRx, Treatment experienced. *P-value 0.05 considered statistically significant.

### Platelet count and coagulation profiles of study participants

In the present study, the median (IQR) of PT, APTT and INR values showed a significant difference across groups. Statistically significant difference was observed in the median (IQR) of PT (P < 0.001), APTT (P = 0.041), and INR (P < 0.001) results between newly diagnosed hypertensive patients and healthy controls. Moreover, there was also a significant difference in PT (P = 0.011), APTT (P = 0.020), and INR (P = 0.012) results between hypertensive patients undergoing treatment and healthy controls. However, the present study was not found a significant difference in any of the coagulation profiles between newly diagnosed and on treatment hypertensive patients (Table 2).

**Table 2:** Comparison of platelet count and coagulation profiles of study participants at Felege Hiwot Comprehensive Specialized Hospital, Bahir Dar, Northwest Ethiopia, 2023, (n = 180).

Parameters	NDx (n = 60)	TRx (n = 60)	Controls (n = 60)	P-values		
	Median (IQR)	Median (IQR)	Median (IQR)	NDx Vs. TRx	NDx Vs. Controls	TRx Vs. Controls
PLT count (10^3^/µl)	256.5 (201.7-296.0)	247.5 (186.2-297.7)	219.5 (197.0-263.0)	0.087	0.087	0.087
PT (sec)	15.0 (13.8-16.4)	14.40 (13.60-16.20)	13.9 (12.9-14.9)	0.325	<0.001*	0.011*
APTT (sec)	34.2 (28.1-39.3)	33.8 (30.2-37.9)	32.1 (29.3-34.4)	0.784	0.041*	0.020*
INR	1.3 (1.2-1.4)	1.2 (1.1-1.4)	1.2 (1.0-1.3)	0.161	<0.001*	0.012*

APTT, Activated Partial Prothrombin Time; NDx, Newly Diagnosed; TRx, Treatment experienced; INR, International Normalized Ratio; IQR, inter quartile range; PLT, Platelet; PT, Prothrombin time, Sec, Second. *P-value 0.05 considered statistically significant.

### Coagulation profile abnormalities among the hypertensive patient

The overall hemostatic abnormality in hypertensive patient was 83 (69.2%). Of these, hemostatic abnormality was observed in 45 (75%) newly diagnosed and 38.0 (63.3%) treatment experienced hypertensive patients. Furthermore, thrombocytopenia, prolonged PT, APTT, and INR was found among 13.0 (10.8), 35.0 (29.2), 51.0 (42.5), and 66.0 (55.0) of the study participants, respectively (Table 3).

**Table 3:** Coagulation profile abnormalities among hypertensive patient participants at Felege Hiwot Comprehensive Specialized Hospital, Bahir Dar, Northwest Ethiopia, 2023, (n = 120).

Parameters		NDx, n (%)	TRx, n (%)	Total n (%)	RR
**PLT count *(10*** ^***3***^ ***/µl)***	No thrombocytopenia	55.0 (91.7)	52.0 (86.7)	107.0 (89.2)	150-450
	Thrombocytopenia	5.0 (8.3)	8.0 (13.3)	13.0 (10.8)	
**PT *(Sec)***	Normal	41.0 (68.3)	44.0 (73.3)	85.0 (70.8)	12-16
	Prolonged	19.0 (31.7)	16.0 (26.7)	35.0 (29.2)	
**APTT *(Sec)***	Normal	33.0 (55.0)	36.0 (60.0)	69.0 (57.5)	30-36
	Prolonged	27.0 (45.0)	24.0 (40.0)	51.0 (42.5)	
**INR**	Normal	24.0 (40.0)	30.0 (50.0)	54.0 (45.0)	0.9-1.2
	Prolonged	36.0 (60.0)	30.0 (50.0)	66.0 (55.0)	
**Overall hemostasis status**	Normal	15.0 (25.0)	22.0 (36.7)	37.0 (30.8)	
	Abnormal	45.0 (75.0)	38.0 (63.3)	83.0 (69.2)	

PLT, Platelet; PT, Prothrombin time; APTT, Activated Partial Prothrombin Time; INR, International Normalized Ratio; Sec, Second; RR, Reference range.

## Discussion

It has been documented that a significant change was observed in platelet parameters and coagulation profiles in hypertensive patients as compared to normotensive individuals [24–28]. In the present study, the overall hemostatic abnormality in hypertensive patient was 83 (69.2%), and significant difference was found in the median of APTT, PT and INR between hypertensive patients as compared to control. The median platelet count was higher in newly diagnosed hypertensive patients as compared to the control group, but the difference was not statistically significant. The findings indicated that newly diagnosed and treatment experienced hypertensive patients exhibited significantly higher median for PT and INR compared to the normotensive controls. This finding was coherent with the finding reported in Nigeria [25, 27, 28], Pakistan [24], Al Gezira state [26] and Khartoum State [40] of Sudan. The higher level of PT and INR among hypertensive patients could be attributed to endothelial damage caused by atherosclerosis, which is commonly associated with hypertension [54]. In addition, hypertension is related to low-grade inflammation along with the influence of inflammatory markers, which has an impact on the coagulation system, leading to prolonged PT and INR values [55].

This study unveiled a statistically significant increase in median APTT value in hypertensive patients compared to their normotensive counterparts. These findings were similar to previously reported results in Pakistan [24] and Nigeria [25]. Several plausible explanations exist for this phenomenon. Chronic high blood pressure might wreak havoc on the endothelium of blood vessels, triggering the release of procoagulant factors and activating platelets, ultimately prolonging APTT. Another culprit could be hyperactive platelets, more prone to clumping together and altering APTT measurements. Additionally, the influence of antihypertensive medications, particularly diuretics and beta-blockers, on coagulation pathways [56]. It’s crucial to acknowledge conflicting evidence presented from Sudan by Merghani [40] and Eman [26] whose studies didn’t detect significant APTT differences between hypertensive and normotensive individuals. This discrepancy could be attributed to several factors, including smaller sample sizes, variations in patient characteristics and co-morbidities, or even methodological differences. Therefore, further research is warranted to solidify the link between hypertension and altered APTT. Ultimately, a comprehensive understanding of this potential association can pave the way for improved strategies in managing and preventing complications associated with hypertension.

Regarding coagulation abnormalities, higher prevalence of coagulation abnormalities among hypertensive participants was observed. The overall hemostatic abnormality in hypertensive patient was 83 (69.2%). Of these, hemostatic abnormality was observed in 45 (75%) newly diagnosed and 38.0 (63.3%) treatment experienced hypertensive patients. The higher prevalence among those newly diagnosed cases compared to on treatment suggests, suggesting potential initial effects and highlighting the increased coagulopathy risk associated with hypertension. High blood pressure in untreated patients can damage the inner lining of blood vessels, which might trigger abnormal platelet activation and coagulation changes. Conversely, antihypertensive medications might help normalize blood pressure, reducing stress on the endothelium and potentially lowering the risk of coagulopathy. Additionally, treating hypertension helps prevent atherosclerosis, further reducing the risk of abnormal clotting [57]. It’s important to acknowledge that other factors like age, co-morbidities, and lifestyle habits can also influence coagulation profiles independently, potentially impacting the observed trends [54, 58].

On the other hand, thrombocytopenia was slightly higher among hypertensive patients receiving treatment compared to those who were newly diagnosed. The possible reason for the high prevalence of thrombocytopenia among those hypertensive patients who are taking anti-hypertensive treatment could be certain anti-hypertensive medications such as thiazide diuretics have been associated with an increased risk of thrombocytopenia as side effects. It can affect the production of platelets in the bone marrow, leading to a decrease in platelet levels and an increased risk of thrombocytopenia [59, 60].

According to our extensive literature, there has been no comprehensive study assessing the coagulation profile of hypertensive patients in Ethiopia, specifically in the study area, which is addressed by the current study. However, a key limitation lies in its study nature, i.e., being a comparative cross-sectional study focusing on the comparison of specific coagulation tests. Additionally, while the study investigates coagulation profiles, its focus on specific tests might have overlooked other potentially crucial aspects of coagulation function, leaving a gap in understanding the full picture. While this study provides a valuable snapshot of associations, further research using longitudinal designs and a wider range of coagulation tests is needed to explore causal relationships between hypertension and coagulation.

## Conclusion and recommendation

In the present study, hemostatic abnormality was observed in majority of hypertensive patients. In addition, there was a statistically significant variation in the APTT, PT, and INR values of hypertensive patients compared to normotensive controls. The platelet count of hypertensive patients is also higher than that of normotensive controls even if it is not statistically significant. Thus, the prediction of the coagulation changes enables the clinician to establish an effective and early therapeutic intervention to prevent the occurrence of major hypertensive complications. Based on the above findings, it is important to assess the changes in coagulation parameters because it may indicate a risk of developing complications before it becomes worse. Further longitudinal cohort studies are recommended to clarify the definitive pathophysiological mechanisms of hypertension regarding coagulation parameters.

## Supporting information

S1 File Questionnaire. (DOCX)

S2 File Raw data (HTN + control anonymized data set). (XLSX)

## Abbreviations

APTT: Activated Partial Thromboplastin Time
BMI: Body Mass Index
BP: Blood Pressure
DBP: Diastolic Blood Pressure
EDTA: Ethylene Diamine Tetra Acetic Acid
FHCSH: Felege Hiwot Comprehensive Specialized Hospital
HIV: Human Immunodeficiency Virus
INR: International Normalized Ratio
PAH: Pulmonary Arterial Hypertension
PPP: Platelet Poor Plasma
PT: Prothrombin Time
SBP: Systolic Blood Pressure

## References

[1] WHO. Global report on hypertension: the race against a silent killer. Geneva: WHO. 2023.

[2] (NCD-RisC), N R F C. Worldwide trends in hypertension prevalence and progress in treatment and control from 1990 to 2019: a pooled analysis of 1201 population-representative studies with 104 million participants. Lancet. 2021;398(10304):957–80. 34450083 10.1016/S0140-6736(21)01330-1 PMC8446938

[3] Mills KT, Stefanescu A, He J. The global epidemiology of hypertension. Nat Rev Nephrol. 2020;16(4):223–37. doi: 10.1038/s41581-019-0244-2 32024986 PMC7998524

[4] Campbell NRC, Lemogoum D. Hypertension in sub-Saharan Africa: a massive and increasing health disaster awaiting solution. Cardiovasc J Afr. 2015;26(4):152–4. 26407216 PMC4683293

[5] Guwatudde D, Nankya-Mutyoba J, Kalyesubula R, Laurence C, Adebamowo C, Ajayi I, et al. The burden of hypertension in sub-Saharan Africa: a four-country cross sectional study. BMC Public Health. 2015;15:1211. doi: 10.1186/s12889-015-2546-z 26637309 PMC4670543

[6] Tesfa E, Demeke D. Prevalence of and risk factors for hypertension in Ethiopia: a systematic review and meta-analysis. Health Sci Rep. 2021;4(3):e372. doi: 10.1002/hsr2.372 34589614 PMC8459032

[7] Gebreyohaness H. Prevalence of hypertension and associated factors among adults in Bahir Dar city northwest Ethiopia. 2020;S(4).

[8] Bowman TS, Sesso HD, Gaziano JM. Effect of age on blood pressure parameters and risk of cardiovascular death in men. Am J Hypertens. 2006;19(1):47–52. doi: 10.1016/j.amjhyper.2005.06.024 16461190

[9] WHO. A global brief on hypertension World Health Day. 2013.

[10] Karabulut A. Clinical implication of hematological indices in the essential hypertension. WJH. 2015;5(2):93. doi: 10.5494/wjh.v5.i2.93

[11] Mancia G, Fagard R, Narkiewicz K, Redón J, Zanchetti A, Böhm M, et al. 2013 ESH/ESC Guidelines for the management of arterial hypertension: the Task Force for the management of arterial hypertension of the European Society of Hypertension (ESH) and of the European Society of Cardiology (ESC). J Hypertens. 2013;31(7):1281–357. doi: 10.1097/01.hjh.0000431740.32696.cc 23817082

[12] Babu R, Kumar N, Solepure A, Shaikh R. Comparison of hematological parameters in primary hypertensives and normotensives of Sangareddy. international journal of biomedical research. 2015;6:309–15.

[13] Abdollahi A, Shoar N, Shoar S, Rasoulinejad M. Extrinsic and intrinsic coagulation pathway, fibrinogen serum level and platelet count in HIV positive patients. Acta Med Iran. 2013;51(7):472–6. 23945892

[14] Aynalem M, Shiferaw E, Gelaw Y, Enawgaw B. Coagulopathy and its associated factors among patients with a bleeding diathesis at the University of Gondar Specialized Referral Hospital, Northwest Ethiopia. Thromb J. 2021;19(1):36. doi: 10.1186/s12959-021-00287-6 34074308 PMC8170961

[15] Chaudhary PK, Kim S, Kim S. An Insight into Recent Advances on Platelet Function in Health and Disease. Int J Mol Sci. 2022;23(11). 10.3390/ijms23116022 PMC9181192 35682700

[16] Hayne OA. Screening tests of the hemostatic system. Can Fam Physician. 1976;22:33–5. 20469270 PMC2378355

[17] Limijadi EKS, Suromo LB, Budiwiyono I. Prothrombine and activated partial thromboplastin time are prolonged in hepatic cirrhosis. Universa Medicina. 2016;35(1):26.

[18] Rad F, Dabbagh A, Dorgalaleh A, Biswas A. The Relationship between Inflammatory Cytokines and Coagulopathy in Patients with COVID-19. J Clin Med. 2021;10(9). 10.3390/jcm10092020 PMC8125898 34065057

[19] Xu C, Song Z, Hu L-T, Tong Y-H, Hu J-Y, Shen H. Abnormal platelet parameters in inflammatory bowel disease: a systematic review and meta-analysis. BMC Gastroenterol. 2024;24(1):214. doi: 10.1186/s12876-024-03305-9 38961334 PMC11221001

[20] Asrat D, Tesfaye G, Gedefaw L, Addisu W, Yemane T. Hemostatic abnormality and associated factors in diabetic patients at Jimma University Specialized Hospital, Jimma, Southwest Ethiopia: A comparative cross-sectional study. Ethiopian Journal of Health Sciences. 2019;29(2):251–8. 31011273 10.4314/ejhs.v29i2.12 PMC6460446

[21] Ebrahim H, Asrie F, Getaneh Z. Basic Coagulation Profiles and Platelet Parameters Among Adult Type 1 and Type 2 Diabetes Patients at Dessie Referral Hospital, Northeast Ethiopia: Comparative Cross-Sectional Study. J Blood Med. 2021;12:33–42. doi: 10.2147/JBM.S287136 33536804 PMC7850412

[22] Aynalem M, Adane T, Getawa S. Magnitude of Coagulation Abnormalities and Associated Factors Among Patients with Heart Diseases at the University of Gondar Comprehensive Specialized Hospital. Vasc Health Risk Manag. 2022;18:617–27. doi: 10.2147/VHRM.S371912 35959111 PMC9362846

[23] Tesfaye G, Yemane T, Gedefaw L. Hemostatic profile and associated factors of hemostatic abnormality in human immunodeficiency virus infected adults attending Jimma University Specialized Hospital, Southwest Ethiopia: A case-control study. Journal of Blood Disorders & Transfusion. 2015;6(6):1–8.

[24] Ali Jiskani S. Prothrombin time (PT), activated partial thromboplastin time (APTT) and international normalized ratio (INR) as predictive factors of coagulopathy in newly diagnosed hypertensive patients. Hematology & Transfusion International Journal. 2017;4(3).

[25] Eledo BE, Izah SC, Okamgba OC. Prothrombin time activated partial thromboplastin time and platelets count among hypertensive patients attending a tertiary health institution Yenagoa, Nigeria. 2018;1:3.

[26] Eman. Estimation of prothrombin time, activated partial thromboplastin time among hypertensive patients in al-gezira state. 2019.

[27] Nnenna Adaeze N, Uchenna Emeribe A, Abdullahi Nasiru I, Babayo A, Uko EK. Evaluation of prothrombin time and activated partial thromboplastin time in hypertensive patients attending a tertiary hospital in calabar, Nigeria. Adv Hematol. 2014;2014:932039. doi: 10.1155/2014/932039 25477963 PMC4248403

[28] Nwovu AI, Obeagu EI, Obeagu GU. Evaluation of platelet and prothrombin time in hypertensive patients attending clinic in federal teaching hospital Abakaliki. 2018;1(5):93–5.

[29] Tabas I, García-Cardeña G, Owens GK. Recent insights into the cellular biology of atherosclerosis. J Cell Biol. 2015;209(1):13–22. doi: 10.1083/jcb.201412052 25869663 PMC4395483

[30] Vrigkou E, Tsangaris I, Bonovas S, Kopterides P, Kyriakou E, Konstantonis D, et al. Platelet and coagulation disorders in newly diagnosed patients with pulmonary arterial hypertension. Platelets. 2019;30(5):646–51. doi: 10.1080/09537104.2018.1499890 30047809

[31] Lip GYBA. Thrombosis and hypertension. J Hum Hypertens. 2000;10(8).

[32] Kazimierczyk R, Kamiński K. The role of platelets in the development and progression of pulmonary arterial hypertension. Adv Med Sci. 2018;63(2):312–6. doi: 10.1016/j.advms.2018.04.013 29885631

[33] Kopeć G, Moertl D, Steiner S, Stępień E, Mikołajczyk T, Podolec J, et al. Markers of thrombogenesis and fibrinolysis and their relation to inflammation and endothelial activation in patients with idiopathic pulmonary arterial hypertension. PLoS One. 2013;8(12):e82628. doi: 10.1371/journal.pone.0082628 24312667 PMC3847115

[34] Nadar S, Lip GYH. The prothrombotic state in hypertension and the effects of antihypertensive treatment. Curr Pharm Des. 2003;9(21):1715–32. doi: 10.2174/1381612033454559 12871204

[35] Rafaqat S, Khalid A, Riaz S, Rafaqat S. Irregularities of Coagulation in Hypertension. Current Hypertension Reports. 2023;25(10):271–86. 37561240 10.1007/s11906-023-01258-0

[36] Vrigkou E, Tsantes AE, Kopterides P, Orfanos SE, Armaganidis A, Maratou E, et al. Coagulation Profiles of Pulmonary Arterial Hypertension Patients, Assessed by Non-Conventional Hemostatic Tests and Markers of Platelet Activation and Endothelial Dysfunction. Diagnostics (Basel). 2020;10(10):758. doi: 10.3390/diagnostics10100758 32992591 PMC7601126

[37] Bazan IS, Fares WH. Hypercoagulability in Pulmonary Hypertension. Clin Chest Med. 2018;39(3):595–603. 30122183 10.1016/j.ccm.2018.04.005

[38] Inanc T, Kaya MG, Yarlioglues M, Ardic I, Ozdogru I, Dogan A. The mean platelet volume in patients with non-dipper hypertension compared to dippers and normotensives. Blood Pressure. 2010;19(2):81–5. 20367545 10.3109/08037050903516284

[39] Lee AJ. The role of rheological and haemostatic factors in hypertension. J Hum Hypertens. 1997;11(12):767–76. doi: 10.1038/sj.jhh.1000556 9468002

[40] Merghani MMHF. Coagulation disturbance among essential hypertensive and diabetes mellitus type II patients - Khartoum state. Bangladesh Journal of Medical Science. 2014;15(03):424–9.

[41] Reriani MK, Lerman LO, Lerman A. Endothelial function as a functional expression of cardiovascular risk factors. Biomark Med. 2010;4(3):351–60. doi: 10.2217/bmm.10.61 20550469 PMC2911781

[42] Vanhoutte PM, Feletou M, Taddei S. Endothelium-dependent contractions in hypertension. Br J Pharmacol. 2005;144(4):449–58. doi: 10.1038/sj.bjp.0706042 15655530 PMC1576026

[43] Sahin I, Karabulut A, Avci II, Okuyan E, Biter HI, Yildiz SS, et al. Contribution of platelets indices in the development of contrast-induced nephropathy. Blood Coagul Fibrinolysis. 2015;26(3):246–9. doi: 10.1097/MBC.0000000000000107 24695089

[44] Arkew M, Yemane T, Mengistu Y, Gemechu K, Tesfaye G. Hematological parameters of type 2 diabetic adult patients at Debre Berhan Referral Hospital, Northeast Ethiopia: A comparative cross-sectional study. PLoS One. 2021;16(6):e0253286. doi: 10.1371/journal.pone.0253286 34125859 PMC8202906

[45] Enawgaw B, Adane N, Terefe B, Asrie F, Melku M. A comparative cross-sectional study of some hematological parameters of hypertensive and normotensive individuals at the university of Gondar hospital, Northwest Ethiopia. BMC Hematol. 2017;17:21. doi: 10.1186/s12878-017-0093-9 29209503 PMC5704458

[46] Sileshi B, Urgessa F, Wordofa M. A comparative study of hematological parameters between hypertensive and normotensive individuals in Harar, eastern Ethiopia. PLoS One. 2021;16(12):e0260751. doi: 10.1371/journal.pone.0260751 34874952 PMC8651120

[47] Gashu K, Gebre-Egziabher T, Maru M. Drivers for urban green infrastructure development and planning in two Ethiopian cities: Bahir Dar and Hawassa. Arboricultural Journal. 2019;41(1):48–63. doi: 10.1080/03071375.2019.1564602

[48] Coulter B. UniCel® DxH 800 Coulter® Cellular Analysis System Instructions for Use. 2009.

[49] Patel S, Gupta S, Patel M, Mahadik J, Patel K, Patel A. A study of coagulation profile in neoplastic conditions. International Journal of Medical Science and Public Health. 2016;5(3):402.

[50] Morgan BL. Understanding power and rules of thumb for determining sample size. 2007;3(2).

[51] Prevention CfDCa. Healthy Weight, Nutrition, and Physical Activity. 2022.

[52] NHANES. Complete blood count with 5-Part Differential laboratory procedure manual. 2014:1-174.

[53] Chandler WL, Laspada AR. Handbook of diagnostic Hemostasis and Thrombosis tests. University of Washington. 2005.

[54] Deanfield JE, Halcox JP, Rabelink TJ. Endothelial function and dysfunction: testing and clinical relevance. Circulation. 2007;115(10):1285–95. doi: 10.1161/CIRCULATIONAHA.106.652859 17353456

[55] Zhang Z, Zhao L, Zhou X, Meng X, Zhou X. Role of inflammation, immunity, and oxidative stress in hypertension: New insights and potential therapeutic targets. Front Immunol. 2023;13:1098725. doi: 10.3389/fimmu.2022.1098725 36703963 PMC9871625

[56] Schrader J. Stroke and hypertension. Internist (Berl). 2009;50(4):423–32. 19308341 10.1007/s00108-008-2291-9

[57] Lee AJ. The role of rheological and haemostatic factors in hypertension. J Hum Hypertens. 1997;11(12):767–76. doi: 10.1038/sj.jhh.1000556 9468002

[58] Junker R, Heinrich J, Schulte H, Erren M, Assmann G. Hemostasis in normotensive and hypertensive men: results of the PROCAM study. The prospective cardiovascular Münster study. J Hypertens. 1998;16(7):917–23. doi: 10.1097/00004872-199816070-00004 9794731

[59] Visentin GP, Liu CY. Drug-induced thrombocytopenia. Hematol Oncol Clin North Am. 2007;21(4):685–96, vi. doi: 10.1016/j.hoc.2007.06.005 17666285 PMC1993236

[60] Lundh B, Hasselgren KH. Hematological side effects from antihypertensive drugs. Acta Med Scand Suppl. 1979;628:73–5. doi: 10.1111/j.0954-6820.1979.tb00782.x 288302

