# Supplementary material for: Platelet count and coagulation profiles of adult hypertensive patients at Felege Hiwot comprehensive specialized hospital, Northwest Ethiopia: A comparative cross-sectional study
Source: PLoS One. 2025 Aug 12;20(8):e0329022. doi: 10.1371/journal.pone.0329022 (PMC12342298; doi:10.1371/journal.pone.0329022)
Supplement: S1 File — (DOCX) [file pone.0329022.s001.docx]

## Questionnaire

Data collection questionnaire designed to conduct a study on the assessment of Platelet count and Coagulation Profiles of Adult Hypertensive Patients at Felege Hiwot Comprehensive Specialized Hospital, Northwest Ethiopia: A Comparative Cross-Sectional Study. **Instructions**: This questionnaire contains a question which is pertinent to the research objectives. You are kindly requested to answer all the questions as much as possible. Does the patient fulfill all inclusion criteria and no exclusion criteria? 1. No 2. Yes

Serial number ___________ Identification number ____________Date _________

A. Data collection format for socio-demographic, anthropometric and clinical data

| Sr. No | Questions | Possible answers |
| --- | --- | --- |
| **Part I. Sociodemographic variables** | | |
| 1 | Age (in years) | _______ |
| 2 | Sex | 1. Male 2. Female |
| 3 | Residence | 1. Urban 2. Rural |
| 4 | Education | 1. Unable to read and write 2. Attend primary school 3. Attend secondary school 4. Attend higher education |
| 5 | Marital status | 1. Single 2. Married |
| 6 | Occupational Status | 1. Student 2. Government employee 3. Private employee 4. Farmer 5. Merchant 6. House wife |
| **Part II. Anthropometric and BP measurements** | | |
| 7 | Weight | _____(KG) |
| 8 | Height | _____(meter) |
| 9 | BMI (weight in Kg/height m^2^) | ______(kg/m^2^) |
| 10 | Systolic blood pressure (SBP) | _______mm/Hg |
| 11 | Diastolic blood pressure (DBP) | _______mm/Hg |
| **Part III. Clinical variables** (for Hypertensive group) | | |
| 12 | Duration of hypertension since diagnosis in years | ________ |
| 13 | Antihypertension drug use? | 1. No 2. Yes |

**B. Data collection format for Laboratory data**

Serial number ___________ Identification number ____________Date _________

| 1 | PLT count | ___________________×10^3^/µl |  |
| --- | --- | --- | --- |
| 2 | Coagulation parameters |  |  |
|  | PT | ______sec |  |
|  | APTT | _______sec |  |
|  | INR | ______sec |  |
